# Early Schizophrenia and Bipolar Disorder Patients Display Reduced Neural Prepulse Inhibition

**DOI:** 10.3390/brainsci12010093

**Published:** 2022-01-11

**Authors:** Rodrigo San-Martin, Maria Inês Zimiani, Milton Augusto Vendramini de Ávila, Rosana Shuhama, Cristina Marta Del-Ben, Paulo Rossi Menezes, Francisco José Fraga, Cristiane Salum

**Affiliations:** 1 Centro de Matemática, Computação e Cognição, Universidade Federal do ABC, São Bernardo do Campo 09606-045, Brazil; rodrigo.san@ufabc.edu.br (R.S.-M.); crisalum@gmail.com (M.I.Z.); 2 Ribeirão Preto Medical School, Universidade de São Paulo, Ribeirão Preto 14040-900, Brazil; milton_avila@usp.br (M.A.V.d.Á.); roshuhama@gmail.com (R.S.); delben@fmrp.usp.br (C.M.D.-B.); 3 Population Mental Health Research Center, Universidade de São Paulo, São Paulo 01246-903, Brazil; pmenezes@usp.br; 4 Department of Preventive Medicine, Faculdade de Medicina, Universidade de São Paulo, São Paulo 01246-903, Brazil; 5 Centro de Engenharia, Modelagem e Ciências Sociais Aplicadas, Universidade Federal do ABC, Santo André 09210-580, Brazil; francisco.fraga@ufabc.edu.br

**Keywords:** sensory gating, electroencephalography, psychosis, schizophrenia, bipolar disorder, EU-GEI, neural PPI

## Abstract

Background: Altered sensorimotor gating has been demonstrated by Prepulse Inhibition (PPI) tests in patients with psychosis. Recent advances in signal processing methods allow assessment of neural PPI through electroencephalogram (EEG) recording during acoustic startle response measures (classic muscular PPI). Simultaneous measurements of muscular (eye-blink) and neural gating phenomena during PPI test may help to better understand sensorial processing dysfunctions in psychosis. In this study, we aimed to assess simultaneously muscular and neural PPI in early bipolar disorder and schizophrenia patients. Method: Participants were recruited from a population-based case-control study of first episode psychosis. PPI was measured using electromyography (EMG) and EEG in pulse alone and prepulse + pulse with intervals of 30, 60, and 120 ms in early bipolar disorder (n = 18) and schizophrenia (n = 11) patients. As control group, 15 socio-economically matched healthy subjects were recruited. All subjects were evaluated with Rating Scale, Hamilton Rating Scale for Depression, and Young Mania Rating Scale questionnaires at recruitment and just before PPI test. Wilcoxon ranked sum tests were used to compare PPI test results between groups. Results: In comparison to healthy participants, neural PPI was significantly reduced in PPI 30 and PPI60 among bipolar and schizophrenia patients, while muscular PPI was reduced in PPI60 and PPI120 intervals only among patients with schizophrenia. Conclusion: The combination of muscular and neural PPI evaluations suggested distinct impairment patterns among schizophrenia and bipolar disorder patients. Simultaneous recording may contribute with novel information in sensory gating investigations.

## 1. Introduction

Patients with bipolar disorder (BP) and patients with schizophrenia (SZ) show overlaps of symptoms and deficits [1,2,3,4,5,6] that challenge the dichotomy proposed in Kraepelin’s original classification of manic-depressive psychosis and dementia praecox [7], or the affective and non-affective psychosis of more recent diagnostic classifications. Both are heritable [8], respond similarly to antipsychotics [9,10], share common genetic causes [11,12], illness course and cognition impairments [13,14,15,16], and there is interchange in diagnosis in a fraction of these patients [17]. Sensory processing impairments seem also to be shared among BP and SZ patients [18], but the extension of this superposition still needs to be clarified.

The most common way of evaluating sensory-motor gating impairments is the muscular prepulse inhibition (PPI) test. This test evaluates the eye-blink startle reflex reduction when a weak Prepulse (PP) stimulus precedes an intense Pulse (P) stimulus by few milliseconds [19,20,21,22,23]. This measure recruits the acoustic startle rapid response pathway, constituted by the cochlear root nucleus, caudal pontine reticular nucleus (PnC), and motoneurons [24]. In parallel, the inferior and superior colliculus and the pedunculopontine tegmental nucleus (PPN) are also activated by the stimuli. As the PnC on the rapid pathway may be inhibited by the PPN in the slower pathway, it has been hypothesized that this slower circuit activation by the PP inhibits the startle response evoked by P, resulting in the PPI phenomenon. Additionally, there are higher order structures, cortico-striato-pallido-pontine (CSPP), which overlap with several impaired structures in patients with psychosis [20,24,25,26,27]. The muscular (eye-blink) PPI effect reflects higher order neural impairments as an indirect measure, thus considered a “pontine portal” to these deficits [20].

Reduction in PPI evaluated by measure orbicularis muscle contraction during acoustic stimulation through the analysis of electromyography (EMG) has been consistently reported in SZ [22,28,29,30,31,32] and euthymic BP [33,34,35,36]. However, PPI evaluation in patients with psychoses is a challenging task, as numerous factors influence its outcome, ranging from ethnicity [37] to drug treatment [38,39,40,41]. Our recent meta-analysis revealed only a moderate effect size for muscular PPI in patients with schizophrenia, with high heterogeneity of results between studies, mainly related with methodological aspects of studies [32].

Patients with BP and SZ also display sensory gating deficits that are evaluated by electroencephalography, through the auditory elicited event-related potential (ERP) components P50, mismatch negativity (MMN), and P300 [42]. In those studies, patients with SZ show larger effect sizes than patients with BP in P50 [43,44,45], MMN [46,47], and P300 amplitude and latency [43,48,49,50,51], when compared to healthy controls. Neural PPI evaluates the neural gating phenomenon in the time window of 100–200 ms, whose latency is not explored by any other sensory gating paradigms [52]. However, neural PPI measurements have rarely been performed due to neural signal contamination resulting from strong muscular contractions elicited by the PPI-test stimuli. Fortunately, recent improvements on signal processing now support signal contamination reduction, allowing neural PPI assessment using electroencephalogram (EEG) alongside with classic eye-blink (muscular) [52]. The neural PPI is a more direct measure than the eye-blink muscular PPI and its psychopathological levels have been associated to eye-blink PPI impairment in SZ [53,54,55,56], but not in BP [34,57]. Thus, the concurrent muscular and neural PPI recording to assess the gating phenomena may be a promising approach to reveal dysfunctions and brain processing differences in BP and SZ patients with psychosis, considering that they occur at different timing and are underlied by different neural circuits [58,59].

In the present study, the primary aim was to assess simultaneously muscular and neural PPI in SZ and BP patients, in early stages of psychosis, and compare with age-sex-education matched healthy subjects. A secondary aim was to evaluate the benefit of using the neural PPI test to investigate sensory gating in patients with psychosis. We hypothesized that neural PPI might be more sensitive to psychopathology levels changes than the traditional test. Finally, another aim of this study was to evaluate psychopathological levels related to psychosis, depression, and mania states concerning to the EMG- and EEG-PPI levels.

## 2. Materials and Methods

### 2.1. Participants

Participants had already been enrolled in the “Schizophrenia and other psychosis Translational Research: Environment and Molecular Biology” (STREAM) project, part of the multinational EU-GEI Project [60,61,62]. In that project, participants were included if they lived in the defined catchment area and presented for the first time in their lives to mental health services due to psychotic episodes. Potential participants were excluded if there was evidence of psychotic symptoms precipitated by an organic cause, or transient psychotic symptoms resulting from acute intoxication, as defined by the ICD-10. Controls came from a population-based sample of individuals without history of contact with mental health services due to psychotic symptoms with sex- and age-distribution similar to the general population. A total of 55 subjects aged between 18 and 40 years were then recruited from that study and participated in the PPI recordings, including 13 patients with SZ, 21 patients with BP, and 21 healthy controls (CT), matched with patients according to gender, age group, and educational level. All participants were evaluated at the Early Intervention in Psychosis Program of the Clinical Hospital of the Ribeirão Preto Medical School, University of São Paulo (HC-FMRP-USP). All tests were carried out by a healthcare professional (clinician, nurse, or psychologist) belonging to the clinic health service team.

Participants signed an Informed Consent Form containing information about the reasons, objectives, procedures, risks, and benefits of the study to which they were invited to participate, and were informed that their participation was voluntary, being able to withdraw at any time without loss to their attendance at the institution. Moreover, participants were guaranteed the right to receive information and to have any questions answered, even though this could affect their willingness to continue participating. They were told that anonymity was ensured, and all provided information would be kept confidential. The local research ethics committee approved the study (process No. 32293214.7.0000.5594/2017).

### 2.2. Clinical Assessment

Severity of psychotic symptoms was evaluated with the Brief Psychotic Rating Scale (BPRS) [63], the Hamilton Rating Scale for Depression (HAM-D) [64] test was used to assess severity of depression symptoms, and the Young Mania Rating Scale (YMRS) [65,66] was used to assess manic symptoms severity. Additionally, at the PPI test session day, participants had their hand preference evaluated by the Edinburgh Handedness Inventory (EHI) [67]. Treatments with atypical and typical antipsychotics, mood stabilizers, and benzodiazepines were also recorded. All participants were assessed at STREAM recruitment day and at the PPI session day by clinicians.

### 2.3. Prepulse Inhibition Test Session

Just after clinical assessment, participants seated on a comfortable chair, were told to stare at a mark in the center of a wall and were informed they would hear strong binaural sounds through headphones during the PPI test. After an acclimation period, two blocks of four-type binaural auditory stimuli were pseudo randomly presented with an inter-stimulus interval of 4–7 s. Each type (P and PP+P with 30, 60, or 120 ms PP-P time) was presented 20 times. Before each block, five pulse-alone (P) stimuli were presented and discarded to avoid discrepant high intensity responses. The Ps were applied with 115 dB intensity and the PPs with 85 dB; both consisting of white noise with virtually instantaneous rise and decay duration of 40 and 20 ms, respectively [68,69,70]. Each test was performed under background (white) noise of 70 dB of intensity, which was also present during the 3-min interval between the first and second blocks. The stimuli intensity was properly calibrated at the beginning of each test day using a decibel meter.

### 2.4. Data Processing

#### 2.4.1. Preprocessing

EEG, EMG, and EOG (electrooculogram) were recorded using a portable device (brand Brain Products, model V-Amp) with 16 dry electrodes. EEG was acquired through a cap (BrainVision Acticap, Brain Products, Gilching, Germany) with 11 scalp electrodes (10–20 system: F7, F3, Fz, F4, F8, C3, Cz, C4, P3, Pz, P4). Two channels were used for EMG measurement, one electrode was reserved for EOG and one more was used as an additional reference. Sampling rate was set at 512 Hz for the recording of all electrophysiological signals. Electrode impedance was kept below 15 kΩ. To register the muscular (EMG) acoustic startle response (ASR), two dry electrodes were placed under the right eye: one of them located 2 cm below the pupil (EMG1) and the other 2 cm below the outer edge (EMG2). EOG was monitored by an electrode placed 2 cm above the center of the left eye, only to facilitate artifact removal (eye blinks) from the EEG signals. The right ear lobe was used as reference. To prevent laterality effects (bias), one electrode was placed on the left earlobe and used as an additional reference. EEG and EOG signals were re-referenced offline to the average of left and right earlobes and the (bipolar) EMG signal was formed by the subtraction of EMG2 from EMG1.

EMG, EOG, and EEG signals were analyzed using the MATLAB software running the EEGLAB package [71] and the SASICA plug-in [72]. All signals were notch-filtered at 60, 120, and 180 Hz to remove power grid interference. Also, to avoid signal delay and distortion, filtering was performed in “zero phase” mode (by reversing the signal and filtering it again). High- and low-pass EMG signal filtering were done with fourth-order Butterworth filters at 24 and 200 Hz cutoff frequencies, respectively, as recommended for PPI studies [73,74]. Although we wanted to remove low-frequency noise from EMG, a greater high-pass cutoff could possibly discard PP responses from the analysis, biasing the data to P alone responses. Therefore, we chose the lower limit of 24 Hz for high-pass cutoff recommendation instead of the higher limit of 32 Hz [74]. The absolute EMG signal envelope was obtained through rectification and low-pass filtering (15.9 Hz). Signals were then divided into periods of 600 ms, from −300 to +300 ms in relation to P stimulus (which occurs at 0 ms). The baseline was calculated by averaging the EMG envelope in the time interval of −50 to 0 ms. In each trial, ASR was measured as the maximum amplitude of the EMG envelope in the 20–120 ms interval.

EEG signal drifting was eliminated using a fourth-order Butterworth high-pass filter with cutoff at 0.25 Hz. In order to obtain the ERPs of each subject, the EEG was segmented from −1000 to 1000 ms in relation to P stimulus. Following, the signal was low-pass (40 Hz) filtered (fourth order Butterworth) and had its baseline corrected according to the average EEG activity in the −650 to −150 ms range. For each channel, trials with amplitudes above 300 μV were automatically removed. Myogenic artifacts were automatically removed using Independent Component Analysis (ICA) available in the EEGLAB toolbox [75]. Artifactual components were automatically identified by the SASICA algorithm [72], with its parameters validated on the control group only. Our previous article, which validated our method to obtain neural PPI on the same control group used in this study, describes this procedure in detail [52], comparing it to other methods used in previous studies [76,77,78].

#### 2.4.2. EMG and EEG Processing

The ASR mean for each experimental condition was calculated as the average of ASR peaks of trials belonging to the same condition on the first block. Subjects were classified as “non-responders” and excluded from the study when the mean amplitude of ASR to P was less than 1 μV. According to this criterion, the number of subjects excluded from the CT, BP, and SZ groups was six, three, and two, respectively. Thus, the final number of participants of this study were respectively 15, 18, and 11 for CT, BP, and SZ groups. Additionally, trial-by-trial exclusion of non-synchronized responses was based solely on the removal of extreme outliers [79] according to the following rules: (i) baseline trial above three SDs (standard deviations) from average baseline; (ii) ASR amplitude higher than average +3 SDs; and (iii) trial onset latency three SDs above average.

For each subject, EEG signal averaging over valid trials was performed separately for the four types of auditory stimulation. Next, N100 and P200 (or N1 and P2) were computed as the negative and positive peak in the 60–165 ms and 165–275 ms range, respectively. The subtraction of N1 from P2 formed the P2-N1 ERP for each of the four stimulus types. Lastly, the resulting ERPs were spatially averaged according to scalp region, with frontal electrodes F3, F4, F7, F8, and Fz forming Favg, central electrodes C3, C4, and Cz averaged to get Cavg and the parietal electrodes P3, P4, and Pz to get Pavg.

Finally, percentage of PPI was computed for both P2-N1 ERP (from EEG) and muscular ASR (from EMG) according to the formula: %PPI = 100 × [1−(P−PP)]/P, where P is the pulse alone response and PP is the PP + P response.

### 2.5. Statistical Analysis

All statistical analyses were performed with the R software, version 3.4.4 [80]. Group characteristics comparison (CT × BP × SZ) were performed using analysis of variance (ANOVA) for age and education years, χ^2^-test for sex and Kruskal–Wallis tests for clinical scores. Significance level was set as 5% for all statistical tests. For the patient groups, pairwise comparisons (BP × SZ) on variables age of onset and treatment start were performed with Student’s *t*-tests. Within-group comparisons using Wilcoxon signed-rank tests were also performed to compare clinical scores (BPRS, HAM-D, and YMRS) at STREAM recruitment and PPI session days. The amplitude (P) and %PPI data (PPI30, PPI60, and PPI120) distribution did not hold normality for the three groups according to visual inspection on skewness and Shapiro–Wilk tests. Therefore, Kruskal–Wallis test was applied for the muscular ASR and the three neural PPI (ERP) scalp regions (Favg, Cavg, and Pavg). When significant group differences were found, one-tailed planned pairwise comparisons were performed with Mann–Whitney/Wilcoxon Rank-sum tests using Bonferroni adjustment based on the number of group comparisons in each measure, i.e., CT × BP, CT × SZ, and BP × SZ. For post-hoc tests, results were considered significant if *p* < 0.05. Kendall correlation was performed separately for each group (CT, BP, and SZ) to assess the relationship between ASR amplitude (P, PPI30, PPI60, and PPI120) and %PPI data (%PPI30, %PPI60, and %PPI120), and also to compare the clinical scores (BPRS, HAM-D, and YMRS) at STREAM recruitment and PPI test session days.

## 3. Results

### 3.1. Demographic and Clinical Data

Demographic and clinical characteristics of the sample are presented in Table 1. Educational levels were significantly lower in SZ when compared to CT [F(2,41) = 3.39, *p* = 0.02]. Participants displayed group statistical differences in symptoms severity measures (BPRS [χ^2^(2) = 24.61, *p* < 0.00001], YMRS [χ^2^(2) = 22.35, *p* < 0.001], HAM-D [χ^2^(2) = 15.67, *p* < 0.001]) at STREAM recruitment day, but not at PPI test day, revealing that patients’ symptoms were controlled at the test day. Use of atypical antipsychotic medication was different between BP and SZ. Mood stabilizers were only administered to BP patients.

### 3.2. Prepulse Inhibition

**Figure 1:**
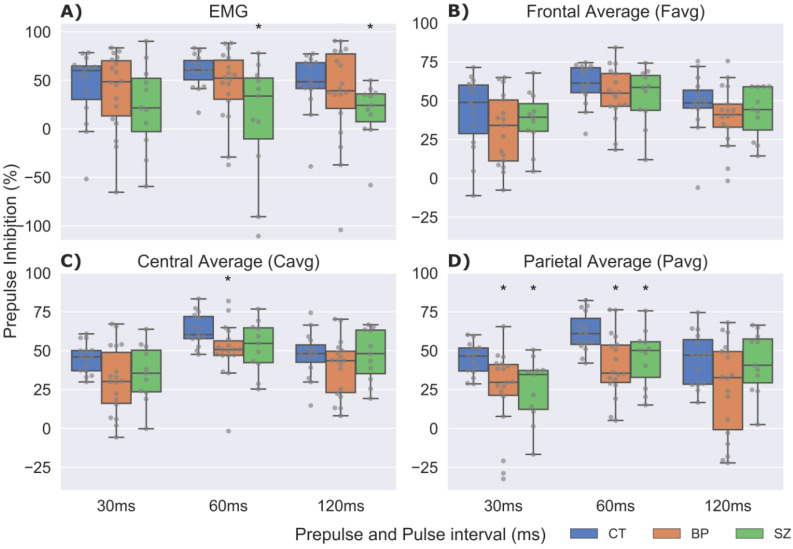
Box- and swarm-plots of muscular and neural P2-N1 PPI for the Prepulse and Pulse intervals of 30, 60, and 120 ms for Control (CT), Manic (BP), and Schizophrenia (SZ) participants. (**A**) Eye-blink startle inhibition; (**B**) Average of the frontal electrodes F3, F4, F7, F8, and Fz; (**C**) Average of the central electrodes C3, Cz, and C4; (**D**) Average of parietal electrodes P3, Pz, and P4. * *p* < 0.05 in the pairwise comparisons for the case groups compared to control groups (CT vs. BP or CT vs. SZ) according to Mann–Whitney/Wilcoxon Rank-Sum Test. Blue boxplots are CT, orange are BP and green represents SZ group. Gray dots are individual %PPI data for each participant. Note that the percentage scale for EMG has a wider range than the neural %PPI.

Median P and PPI values are reported on Table 2. The muscular ASR to P, evaluated by the EMG, was not statistically different between groups [χ^2^(2) = 0.6; *p* > 0.05]. Similarly, neural ASR to P evaluated by P2-N1 was not different between groups at any scalp locations: Favg [χ^2^(2) = 2.9; *p* > 0.05], Cavg [χ^2^(2) = 3.7; *p* > 0.05] nor Pavg [χ^2^(2) = 5.3; *p* > 0.05]. Regarding muscular %PPI (Figure 1A), CT values were significantly higher than SZ for %PPI60 [W(1) = 132; *p* = 0.014] and %PPI120 [W(1) = 137; *p* = 0.006].

As for neural %PPI measured by Cavg (Figure 1C), CT displayed significantly higher %PPI than BP for the %PPI60 [W(1) = 212; *p* = 0.007]. Also, when measured by Pavg (Figure 1D), CT displayed significantly higher %PPI than BP for %PPI30 [W(1) = 208; *p* = 0.011] and %PPI60 [W (1) = 221; *p* = 0.002]. Accordingly, CT exhibited significantly greater %PPI than SZ for %PPI30 [W(1) = 133; *p* = 0.012] and %PPI60 [W(1) = 125; *p* = 0.041].

Kendall correlation analysis on BP group showed a negative association between the HAM-D Score at recruitment day and muscular %PPI for 30 [Kendall’s tau = −0.47; *p* = 0.01], 60 [Kendall’s tau = −0.51; *p* < 0.01], and 120 ms [Kendall’s tau = −0.47; *p* = 0.01] P + PP intervals. In contrast, for the same BP group, neural %PPI60 evaluated by Favg was positively correlated with HAM-D score at recruitment day [Kendall’s tau = 0.52; *p* < 0.01]. Still for BP patients, the YMRS was negatively correlated with neural %PPI120 measured by Favg [Kendall’s tau = −0.63; *p* < 0.01].

**Figure 2:**
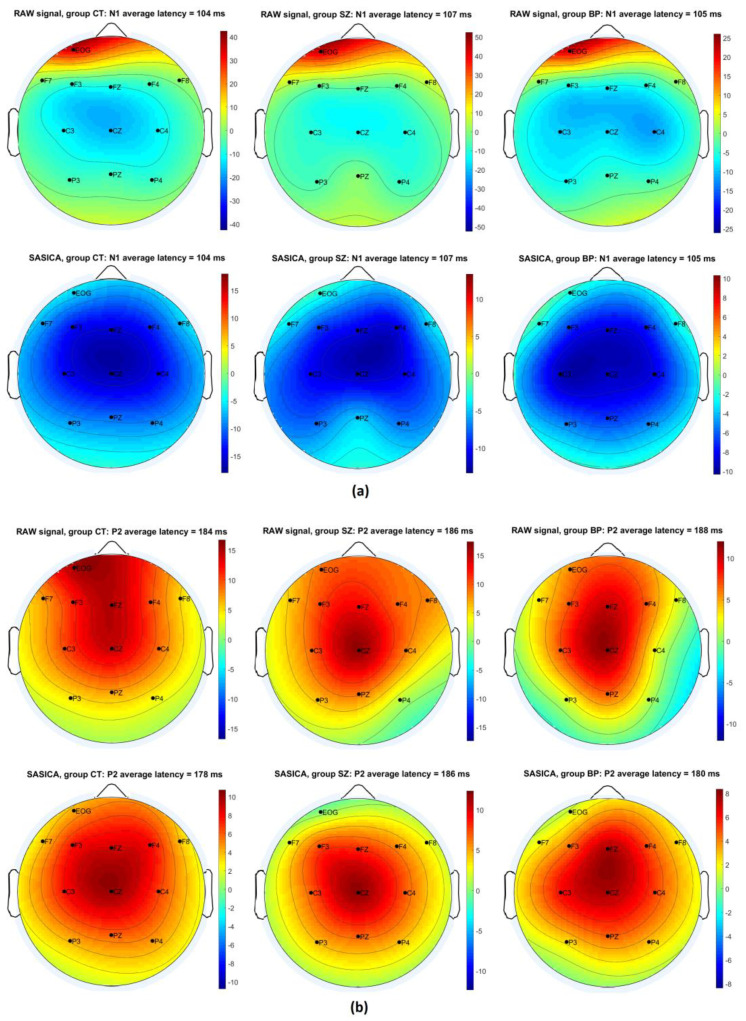
2D topography (grand average ERPs) for the pulse alone (P) condition at latencies N1 (**a**) and P2 (**b**) from CT, SZ, and BP groups before (RAW signal) and after (SASICA) artifacts removal. 2D topography includes Frontal (F7, F3, Fz, F4, and F8), Central (C3, Cz, and C4) and Parietal (P3, Pz, and P4) channels. Eye-blink artifacts due to startle reflex affect mainly the N1 component (RAW signal). After the artifacts’ removal (SASICA), the N1 component displays normal negative activation (blue color) across all scalp channels.

**Figure 3:**
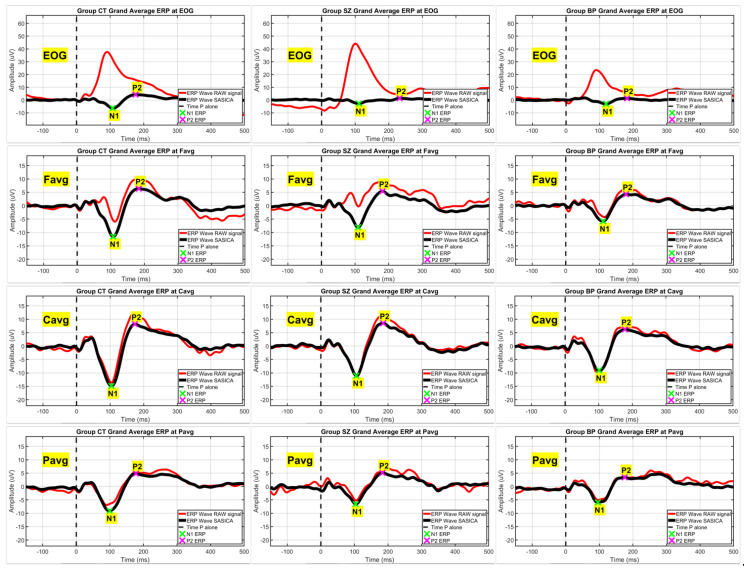
Grand averaged waveforms of all groups before (red line) and after (black line) artifacts’ removal on the EOG electrode and on the average of frontal (Favg), central (Cavg) and parietal (Pavg) channels (lines of subfigures). After artifact’s removal, the N1 and P2 ERPs (green and magenta crosses, respectively) were identified even on the EOG channel, due to the underlying brain activity captured by this electrode, visible only after complete removal of eye-blinks.

**Figure 4:**
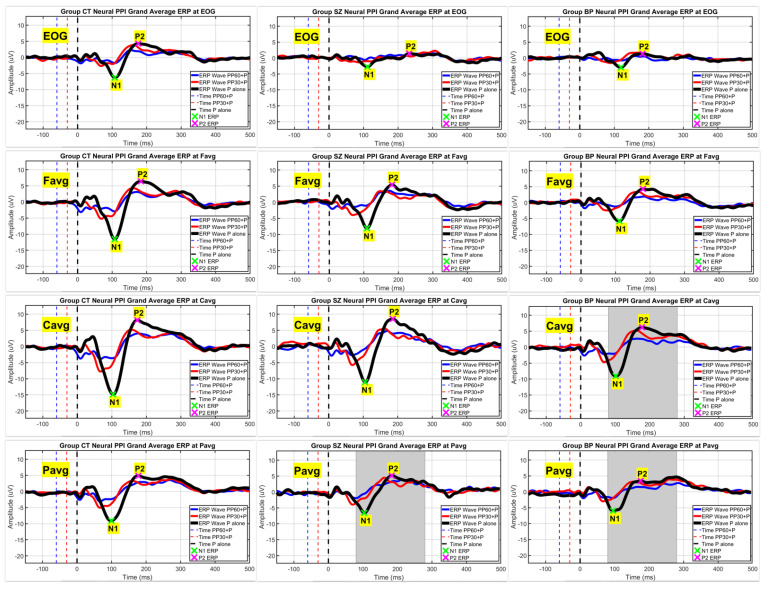
Grand averaged waveforms (after artifacts removal) of from CT, SZ, and BP groups (column subfigures) for the conditions pulse alone (P) and PP + P (30 and 60 ms) at EOG, Favg, Cavg, and Pavg (line subfigures). The N1 and P2 ERPs (green and magenta crosses, respectively) were signalized only for the pulse P condition (black lines), but grand averaged waveforms were also displayed for the PP + P conditions at Prepulse-Pulse intervals of 60 and 30 ms (blue and red lines, respectively), which were the only conditions were significant differences (highlighted in grey color) with the CT group were found in the neural PPI.

Figure 2 and Figure 3 show the efficiency of the SASICA algorithm [72] to eliminate the eye-blinks and other artifacts from the RAW signal. Figure 2 shows the topography (grand average ERP) for the pulse alone (P) condition at latencies N1 and P2 with eye-blink artifacts occurring due to startle reflex affect mainly the N1 component (RAW signal). After artifacts removal (SASICA), the N1 component displays normal negative activation across all scalp channels. In Figure 3, it is possible to observe that eye-blinks were completely eliminated from the EOG channel, enabling the identification of the N1 and P1 ERPs even in this electrode, due to the underlying brain activity captured by EOG. Figure 4 displays the grand averaged waveforms from CT, SZ, and BP groups for the conditions pulse alone (P) and PP+P (30 and 60 ms) at EOG, Favg, Cavg, and Pavg (grey areas signalize statistically significant differences with the control group in neural PPI).

## 4. Discussion

To the best of our knowledge, this is the first time muscular and neural PPI impairments were evaluated in early BP and SZ. We found that only SZ, but not BP patients, presented sensorimotor gating reduction in muscular %PPI at 60 and 120 ms PP + P intervals. In addition, we observed reduction in neural %PPI for both BP and SZ patients. These findings suggest that the evaluation of neural PPI may detect gating impairments in a broader manner than the classical muscular PPI paradigm [52].

In classical eye-blink (muscular) PPI, some previous SZ sensorimotor gating investigations found impairments for the 60 ms but not for the 120 ms PP + P interval [22,53,81], while others observed just the opposite [82,83]. Some studies found %PPI reduction in SZ at both 60 and 120 ms intervals [84,85], but others did not detect any impairment at 60 or 120 ms intervals [86,87]. The moderate effect size of this phenomenon and methodological aspects that may influence the outcomes probably explain such heterogeneity, as we showed in our recent meta-analysis [32]. We controlled several of these factors by including CT participants that were socio-economically matched to patients. Additionally, the PPI test session was performed on average one year and eight months after a patients’ first psychotic episode. Drug treatment after the first episode may have reduced the effect size of %PPI impairments at PPI test session day [69]. The sensorimotor gating impairments using EMG (muscular) were not observed in BP patients. A previous study with BP patients found %PPI reduction in some subgroups, such as males with high depression levels, but did not show abnormalities in female and euthymic BP groups [33], as well as in manic or mixed groups [57]. In contrast, another investigation described %PPI reduction at 60 and 120 ms PP + P intervals [34].

In this study, the neural %PPI measured by the P2-N1 ERP at the parietal region detected reduction in sensory gating in both BP and SZ groups. Muscular and neural PPI recruit different brain regions, with muscular PPI revealing CSPP circuitry alterations through motor output (detected by the EMG signal) and neural PPI directly reflecting neuronal synchronized firings (detected by the EEG signal). They also occur at different temporal scales. Muscular PPI involves the fast ASR and slower PPI modulation circuitry [24], usually detected at 20–120 ms, not necessarily requiring higher order cortical systems activation. Neural PPI is observed on a distinct temporal scale, with the ERPs N1 and P2 occurring, respectively, 100 and 200 ms after stimulus onset and necessarily involves cortical regions. In neural PPI, the (auditory) N1 is time-locked to the stimuli onset and indicates that its perception reached the auditory cortex region [88]. The auditory N1 in response to P alone stimuli is related mainly to the primary and secondary auditory cortices [88]. Differently, the P2 wave has been reported to be more widely distributed within the brain, with two main sources identified, one in the auditory cortex and the other in the frontoparietal region, as well as other weaker sources in the anterior cingulate cortex and the insula [89]. Furthermore, an auditory inhibition paradigm evaluated by magnetoencephalography recording proposed that the N1m potential source is at the lateral side of the transverse gyrus or superior temporal gyrus [90,91]. The source of N1 and P2 were also estimated in a neural PPI paradigm investigating healthy individuals [92] with low resolution brain electromagnetic tomography [93]. The N1 generating source was estimated in the frontal lobe of the right hemisphere, while the source of P2 was estimated in the right upper parietal lobe. In summary, although methodological differences may play a role in these results, the N1 and P2 source estimation investigations suggest different generating origin for the ERPs according to their inhibition state. Moreover, the inhibited N1 and P2 sources are clearly distinct, indicating that the sensory gating is not constrained to one brain region. Hence, the extension of the classical eye-blink (muscular) PPI with concurrent EEG recordings enabling the evaluation of neural PPI, similarly to the PPI and P50 sequential recording [94,95], adds a significant piece of information to the classical PPI research. Nevertheless, this result should be interpreted with caution, as the detection of %PPI impairments on the same electrodes does not directly imply that deficits occur in the same brain structures for both patient groups. While EEG is recorded with high temporal resolution and can be considered a neuroimaging tool, and with some advanced techniques being even able to detecting source localization of brain signals [96,97], it still displays a lower spatial resolution than other techniques such as magnetic resonance imaging (MRI) and positrons emission tomography (PET).

We found a negative correlation between muscular %PPI and the HAM-D score for all PP + P intervals at recruitment day only, not at PPI test session day. Several studies investigating the correlation between symptoms’ severity, as measured with scales such as BPRS/PANSS, YMRS and/or Ham-D, and %PPI levels in a wide range of mental illnesses, as Alzheimer [98], BP [33,99], and SZ [56,83,100,101,102,103,104], did not find significant associations. A small number of studies observed significant correlations between those measures in SZ patients subgroups or for some of the investigated PP + P intervals, such as the PANSS positive or SAPS being negatively correlated to %PPI60 [53,105] and %PPI120 [106]. In the present study, we did not find correlations between BPRS, YMRS or HAM-D scores, and %PPI levels in the SZ patients. It is possible that the neural %PPI, being a more direct measure, would be more sensitive to correlation measures than muscular %PPI, but again we observed no significant correlation between psychopathological scores and the neural %PPI in any of the three electrode averages for the SZ patients. To sum up, as sensory gating belongs to automatic processing domains and clinical symptoms are revealed in more complex domains, association between those measures may not be prone to be reliably detected [100,107,108].

The present study has some limitations. First, 22% of SZ and 45% of BP patients were treated with benzodiazepines. This class of medications is known to reduce %PPI levels among healthy individuals in a dose-dependent pattern [109], a limitation that is recurrent among PPI studies, especially in first-episode psychotic patients [100,110,111], with some studies attenuating possible medication effects by restricting the use of such drugs at the PPI test day [54]. We did not restrict inclusion due to the use of any drug, and all schizophrenia and bipolar patients were under antipsychotic, mood stabilizer, or both treatments, and some were prescribed benzodiazepines, but doses were not recorded in this work. Second, in our study, the limited number of subjects in each group thwarted additional analyses that might have provided more information on sensory gating by subgroups, such as sex [112], antipsychotic drug types [113], and severity of psychotic symptoms [114]. Third, similarly to previous neural PPI studies [76,77,115], this study did not evaluate %PPI on N1 and P2 potentials, separately. The P2-N1 ERP complex is originated in distinct brain regions, therefore biological validity is reduced with this unified analysis. Fourth, we analyzed the signal in the 1–40 Hz range (full band). Sub-band analyses might provide further information [76], as patients’ groups may display alterations limited to specific bands. For example, SZ patients have dysfunctions in resting-state high gamma frequencies when compared to healthy people [116], so it is possible that they also present alterations in the PPI when investigated in specific bands. Fifth, frontal lobe plays a major role in inhibition [117,118], but no group differences were detected in this region. The frontal electrodes were the most contaminated by eye-blink artifacts, and the cleaning process may have attenuated the signal in these regions [72]. Sixth, there are several distinct protocols of PPI test, including different intensities, duration, and source of stimuli; herein, we opted to use a set up similar to those used in many studies with schizophrenia and/or bipolar patients [22,32,36,95] and those used in our previous study [52]. Seventh, we did match cases and controls for potential confounding variables (gender, age group, and educational level), but did not control for potential confounding of smoking status. Lastly, the small sample size may have increased the chance of type II errors, which means not enough statistical power to detect the distinct effects for %PPI in BP and SZ populations. On the other hand, multiple statistical comparisons may have increased the chance of type I errors.

## 5. Conclusions

As implications for future studies, the combination of muscular with neural PPI has the potential to contribute to differential diagnosis after the psychotic outbreak, as different patient groups may display changes in specific electrode regions, band frequencies, or PPI modalities (neural or muscular). Future studies should include larger BP and SZ groups and evaluate other disorders, to assess the predictive validity of combined muscular and neural PPI.

## Figures and Tables

**Table 1:** Socio-demographic and clinical characteristics of the sample.

Characteristics	Control (*n* = 15)	Bipolar (*n* = 18)	Schizophrenia (*n* = 11)	Test Types	Omnibus Statistic and Significance Pairwise Comparisons
Age at STREAM (years)	26.8 ± 7.42	25.55 ± 6.87	27.36 ± 8.93	a	F(2,41) = 0.22, n.s.
Education (years)	12.5 ± 2.69	10.79 ± 2.48	10.02 ± 2.37	a, t	F(2,41) = 3.39, *p* = 0.02; θ
Sex (m/f)	9/6	10/8	8/3	c	χ^2^(2, 41) = 0.87, n.s.
Age of onset (years)	-	24.47 ± 6.84	25.65 ± 8.96	b	t(17.10) = 0.38, n.s.
PPI test—treat. start (years)	-	1.57 ± 0.8	1.84 ± 0.92	b	t(19.01) = 0.81, n.s.
Edinburgh (score)	17.26 ± 3.15	17.94 ± 2.33	17.54 ± 2.38	d	k^2^(2) = 0.50, n.s.
BPRS STREAM (score)	0.93 ± 2.84	9 ± 5.58	12.81 ± 6.86	k, w	k^2^(2) = 24.61, *p* < 0.00001; θ, δ
BPRS at PPI test (score)	1.93 ± 2.65	3.77 ± 7.05	7.45 ± 8.06	k	k^2^(2) = 4.05, n.s.
Hamilton D at STREAM (score)	1.86 ± 5.16	5.27 ± 6.02	9.18 ± 5.84	k, w	k^2^(2) = 15.67, *p* < 0.001; θ, δ
Hamilton D at PPI test (score)	1.93 ± 3.03	2.5 ± 3.09	6.27 ± 6.73	k	k^2^(2) = 5.45, n.s.
YMRS at STREAM (score)	0.53 ± 1.18	13.5 ± 10.18	5.18 ± 4.91	k, w	k^2^(2) = 22.35, *p* < 0.001; θ, δ
YMRS at PPI test (score)	0.53 ± 0.83	3.05 ± 5.77	2.9 ± 3.83	k	k^2^(2) = 2.75, n.s.
Atypical antipsychotic (%)	-	39%	82%	c	χ^2^(1, 29) = 5.09, *p* = 0.05; β
Typical antipsychotic (%)	-	6%	18%	c	χ^2^(1, 29) = 1.17, n.s.
Humor stabilizer (%)	-	61%	-	c	χ^2^(1, 29) = 10.83, *p* < 0.01; β
Benzodiazepines (%)	-	22%	45%	c	χ^2^(1, 29) = 1.72, n.s.

Sex is displayed as the absolute counts for male (m)/female (f) participants. Drug treatment are displayed as the percentage of use in relation to participants of the same group. The remaining characteristics are displayed as the mean ± the standard deviation. Age is displayed at STREAM recruitment day. Brief Psychotic Rating Scale (BPRS), Hamilton D and Young Mania Rating Scale (YMRS) are displayed at STREAM recruitment day and at PPI test day. Statistical Test Types are indicated as: a—ANOVA (CT × BP × SZ); t—Welsh Two Sample t-test (CT × BP) or (BP × SZ) or (BP × SZ); c—χ2 (CT × BP × SZ) or χ2 (BP × SZ); k—Kruskal–Wallis test (CT × BP × SZ); w—Wilcoxon rank sum test pairwise group comparisons. Statistically significant post-hoc pairwise comparisons: θ—CT × BP; δ—CT × SZ; β—BP × SZ.

**Table 2:** Medians of Acoustic Startle Response (ASR) and percentage Prepulse Inhibition for Control, Schizophrenia, and Bipolar Disorder participants.

Electrode	Response	Control (*n* = 15)	Bipolar (*n* = 18)	Schizophrenia (*n* = 11)	Omnibus Statistic
EMG	P Amp (µV)	2.23	1.87	3.04	χ^2^(2) = 0.62, n.s.
	%PPI30	60.05	48.49	21.61	χ^2^(2) = 1.9, n.s.
	%PPI60	60.52	52.25	**33.81**	χ^2^(2) = 6.71, *p* = 0.03
	%PPI120	48.51	39.29	**24.26**	χ^2^(2) = 6.81, *p* = 0.03
Favg	P Amp (μV)	21.89	16.90	13.95	χ^2^(2) = 2.94, n.s.
	%PPI 30	48.89	34.14	39.36	χ^2^(2) = 2.6, n.s.
	%PPI 60	61.36	54.90	58.55	χ^2^(2) = 1.95, n.s.
	%PPI 120	48.52	41.01	44.28	χ^2^(2) = 2.86, n.s.
Cavg	P Amp (µV)	25.26	20.97	19.47	χ^2^(2) = 3.68, n.s.
	%PPI 30	45.96	30.12	35.50	χ^2^(2) = 5.11, n.s.
	%PPI 60	60.29	**50.72**	54.72	χ^2^(2) = 7.46, *p* = 0.02
	%PPI 120	48.14	43.58	48.06	χ^2^(2) = 2.28, n.s.
Pavg	P Amp (µV)	19.70	14.79	13.65	χ^2^(2) = 5.26, n.s.
	%PPI 30	46.52	**29.62**	**34.65**	χ^2^(2) = 9.45, *p* < 0.01
	%PPI 60	61.00	**35.55**	**50.15**	χ^2^(2) = 10.45, *p* < 0.01
	%PPI 120	47.09	32.62	40.59	χ^2^(2) = 3.29, n.s.

In bold, are indicated Schizophrenia and/or Bipolar patients with significantly lower %PPI than the Control group revealed by pairwise (CT × BP or CT × SZ) Mann–Whitney/Wilcoxon Rank-sum tests.
